# Identification of research priorities for suicide prevention in Nepal: a Delphi study

**DOI:** 10.1186/s12888-022-04074-z

**Published:** 2022-06-25

**Authors:** Elisha Joshi, Santosh Bhatta, Sunil Kumar Joshi, Julie Mytton

**Affiliations:** 1 Nepal Injury Research Centre, Kathmandu Medical College Public Limited, P O Box 21266, Kathmandu, Nepal; 2 Centre for Public Health and Wellbeing, University of the West of England, Bristol, UK; 3 Faculty of Health and Applied Sciences, University of the West of England, Bristol, UK; 4 Department of Community Medicine, Kathmandu Medical College Public Limited, Kathmandu, Nepal

**Keywords:** Research priorities, Suicide prevention, Delphi method, Developing countries

## Abstract

**Background:**

Suicide is a significant public health concern in Nepal and there is a need for an evidence-based suicide prevention programme to facilitate stakeholders working towards suicide prevention in Nepal. Collaborative research between stakeholders focussing on shared priorities can help to prevent and control suicide. Hence, we aimed to develop a consensus list of research priorities for suicide prevention in Nepal.

**Methods:**

The Delphi expert consensus method was used to elicit the prioritized research questions for suicide prevention in Nepal. Participants comprised suicide prevention experts (psychologists, psychiatrists, psychiatric nurses, researchers and advocates) and people with lived experience. Three rounds of Delphi were conducted; round 1: one to one interviews involving open ended questions used to generate research questions; round 2: ranking of the research questions using a 5-point Likert scale, and round 3: re-ranking of research questions in light of individual and group responses.

**Results:**

Forty-two participants participated in round 1 followed by 38 in round 2 and 39 in round 3 . 522 research questions were generated through round 1 which were grouped together and reduced to 33 research questions sent for ranking in round 2. Using a cut off of at least 70% of the panel ranking questions as ‘very important’ or ‘important’, 22 questions were retained. These research questions were sent for re-rating in round 3 resulting in a final list of prioritized questions.

**Conclusions:**

This is the first expert consensus study to identify the top research priorities for suicide prevention in Nepal, and used experts in suicide prevention and those with lived experience. A consensus was reached regarding the studies needed to improve suicide data quality, assess the burden and identify factors associated with suicide. A priority driven approach to suicide prevention research may ensure that the research endeavour provides the most useful information for those whose day-to-day work involves trying to prevent suicide.

**Supplementary information:**

The online version contains supplementary material available at 10.1186/s12888-022-04074-z.

## Introduction

Suicide is a global health challenge claiming the lives of almost 800,000 people every year, equivalent to a person dying every 40 s due to suicide [1]. Ranked as the fourth leading cause of mortality among 15–29 year-olds, 77% of the burden of suicide occurs in low- and middle-income countries (LMICs) [2]. Consisting of 11 LMICs, the World Health Organisation South- East Asia region has suicide rates that are higher than the global average (10.2 per 100,000 compared with 9.0 per 100,000) [2]. While, data on suicide and prevention strategies for suicide are scarce in LMICs [3] generally, progress has been made in India with the development of a national suicide prevention strategy [4]. Social stigma and taboos, religious and cultural issues, inadequate reporting systems, scarce resources to assist people who are suicidal all contribute to the substantial public health challenge currently posed by suicide in these settings [5].

Nepal, like many other LMICs, lacks reliable data on suicide and attempted suicide, instead relying on extrapolations from police reports [6]. Estimated suicide rates in Nepal vary widely; a 2014 scoping review projected a suicide rate of 8.6 per 100,000 population, WHO modelled age-standardized suicide rates shows downward trend rates; 24.9 per 100,000 in 2014 [7], and 9.8 per 100,000 in 2019 [8]. In contrast, a five- year study of Nepal police records of suicidal deaths between 2015 and 2019 showed an increase of 33% in suicidal deaths over five years [9]. According to a recent community-based study, self-harm and assault accounted for 44 of the 67 total injury deaths (66%) in two wards in Makwanpur, Nepal [10], despite in contrast to a study using global burden of disease which suggest that only a small proportion (1.6%) of deaths are intentional [11].

Studies estimating the burden of suicide often comprise small hospital-based case series, using data from post-mortem reports of suicide cases [12]. A scoping review of literature on self-harm and suicide behaviour in Nepal found that the victims were predominantly females, belonging to younger age groups, often with mental illness and psychosocial stressors. Hanging and organophosphorus poisoning accounted for more than 90% of suicides in this review [13]. Most of the studies concluded there is shortage of reliable, representative and nationwide data on the burden of suicide in Nepal which was a consequence of limited research [6, 14]. Reasons for the lack of research may include the multifactorial nature of suicide [15], the sensitive nature of the topic, the associated stigma and legal implications [16] and the lack of consensus about where research efforts should be focussed [17]. Mental health experts and non-governmental organizations (NGOs) have spearheaded some suicide prevention programs, such as mental health training for primary health care workers based on the Mental Health Gap action programme, and the initiation of 24-h suicide hotline support services [6] but all these are at the inception stage. Despite the fact that suicide is a serious public health concern in Nepal, the country lacks a national suicide prevention strategy [18]. In light of Nepal’s growing suicide problem, it is imperative to identify and understand stakeholder perspectives and to identify priority research areas in order to inform policy and practice in relation to suicide prevention [17].

Prioritization exercises in health research are intended to assist researchers, funders and policy makers in effectively identifying research with the greatest potential for public health benefit [19]. Suicide prevention strategies developed with a ‘one-size-fits-all’ approach have been found to have limited effectiveness [20] and identifying the areas of most need and gaps will safeguard against duplication and determine immediate and feasible actions [21]. For greater relevance, a priority setting exercise designed to capture a broad range of views is essential. The Delphi methodology has been widely used to achieve group consensus through a series of “rounds” gathering information from a number of stakeholders. This approach has been successfully used for identifying mental health priorities [22, 23]; but seldom for establishing suicide prevention research priorities [17]. Coordinated efforts between experts of various sectors are needed to initiate a national suicide prevention program in Nepal comprised of designing, implementing and testing of economically feasible, evidence based and socio-culturally appropriate suicide prevention strategies [13]. With the aim of underpinning and informing this process with appropriate research, this study used a Delphi approach to identify and generate list of research questions relevant to suicide prevention in Nepal.

## Methods

### Aim

This study aimed to establish a consensus list of potential research questions to facilitate suicide prevention in Nepal, using experts in the field as well as those with lived experience.

### Design

The Delphi method is an multistage iterative process for reaching consensus among a defined group of individuals [23]. Studies have found this technique to be effective when evidence about a phenomenon is known to be limited or incomplete [24]. The Delphi method is based on the premise that the opinions of a group of people outweigh those of an single person, and hence any agreement reached can be considered valid expert opinion [18]. The method consists of a structured process involving a series of ‘Rounds’ where the participants generate, prioritise and re-prioritise potential research areas using feedback from previous rounds. Responses to each round are analysed to produce a final consensus list [18]. Although up to six rounds of Delphi have been known, it is becoming more common for two to three rounds to be the maximum application, depending on the individual study [25]. We determined, a priori, to conduct a three -round Delphi study, comprising an initial round to generate a ‘long list’ of topics, a ranking evaluation in the second round, and a re-ranking in the third round.

### Participants

There are no universally agreed criteria for the selection of experts for a Delphi study [26]. However, when setting research priorities for health conditions, there is a recognised need to include clinicians, researchers, patients and significant others who have experienced the condition of interest. The inclusion of such a diverse group of stakeholders promotes wide-ranging ownership of the research priorities [27]. Hence, this study recruited participants into four panels: academic researchers, practice-based experts, patients and advocates. Purposive and snowball sampling were used to recruit the participants. Including the participants with knowledge and interest in the topic helped to ensure content validity of the study [28]. The detailed eligibility criteria for the participants are given below (Table 1).

**Table 1:** Eligibility criteria for the participants

Participant group	Eligibility to be a participant
Academic researchers	Lead author or co-author in at least one article related to suicide and suicide prevention in Nepal published after 2010, living in Nepal or outside Nepal
Practice based experts (psychiatrists, psychiatric nurses or psychologists)	At least 6 months experience in field relevant to suicide prevention in Nepal and are currently living in Nepal
Patient experts (Survivors of suicide attempts, their care givers or family members)	People living in Nepal who had attempted suicide, at least 6 months previously, were under follow up by the psychiatric department of Kathmandu Medical College and were considered clinically stable, or, their care givers or family members
Advocates	At least 6 months experience in working in non-governmental or governmental organization for suicide prevention in Nepal and are currently living in Nepal

### Recruitment process

We identified potential participants through the following routes. For academic scholars, the corresponding authors of scientific publications reporting suicide in Nepal since 2010 were identified. Practice based experts included psychiatrists, psychologists, and psychiatric nurses who were approached after contacting the Departments of Psychiatry of several medical institutions. The potential participants were given information about the study and were given the opportunity to ask questions. Practice based experts who agreed to take part in the study were asked if any of their colleagues and peers should also be invited to participate (i.e., snowball-sampling). To identify advocates, we approached organizations working towards suicide prevention. Interested participants were asked about other similar organizations working in Nepal. To recruit patient experts and their families to the study, we worked closely with the psychiatric department of Kathmandu Medical College (KMC) to identify and approach patients who have previously attempted suicide and were currently attending the out-patient department. The clinical team only suggested patients who were clinically stable and potentially suitable for inclusion in the study. Only patients who had attempted suicide 6 months, previously could communicate in the native language (Nepali), and were not considered to be currently severely depressed or anxious were identified by the clinical team. To safeguard patient participants, the psychiatric team at KMC were available to provide support if any became distressed during participation, and the department provided facilities (e.g., ventilated, private space) to limit the risk of Covid-19 transmission for both patient and researcher during their meetings.

To inform patients about the study, face to face meetings were set up after the patient’s scheduled follow up appointment in the out-patient department of KMC. There, letters of invitation along with an information sheet were provided. The information sheet advised the potential participants of the purpose of the study, the likely number of rounds, confirmation that participation was voluntary and provide assurances of confidentiality and the opportunity to withdraw at any time. A convenient date and time for the Round 1 interview was scheduled for patients and family members who wished to take part in the study. Potential participants from other expert groups were contacted in accordance with their own preferences. An invitation to participate and additional information about the study were communicated either by phone or online. Potential participants who did not respond and those who declined to participate took no further part in the study. Before commencing with Round 1 interview, written consent was obtained from participants joining face to face interviews, and verbal consent was taken from those participating by phone or online. While taking consent before round 1, participants were given the choice of being contacted online or by phone for round 2 and round 3. All the patient/caregiver participants chose to be contacted via phone, whilst other participants chose online.

### Data collection and analysis

#### Round 1

In round 1 of the study, the participants were given a choice about how they wanted to participate (face to face, online or telephone interview). During each interview, an experienced qualitative researcher (EJ) read out the questionnaire and the participants’ responses were audio recorded with permission (Additional file 1: Annex 1: Questionnaire). Open ended questions were used to encourage participants to express their views on topics, themes or questions they believed were research priorities based on their experience and knowledge [29]. The interviews lasted 15–30 min and data collection for all rounds took place between December 2020 and February 2021. Demographic data on age, gender and years of experience in the field were collated for all the participants.

Professional participants and advocates were asked about their views on the status of suicide and its risk factors in Nepal, together with aspects of suicide prevention that were working well or were less effective. Participants were asked whether they needed any more evidence to better understand suicide or its prevention and to articulate up to 10 areas of research that would help improve suicide prevention. Patients and family members were asked for their views about what could be done by doctors, researchers and at individual, community, or national level to reduce or prevent suicide. To limit the risk of distress in patients, questions were confined to how to best to support those who are considering ending their life. At no stage were patients asked about their own suicide attempt or the contributory factors. Suicide attempt survivors, or their family members’ interviews, were conducted in the hospital out-patient department where clinical support was readily available if required. We had a distress protocol in place, in which the interview would be paused if someone became distressed.Then patient participants or their carers would be offered the choice of either continuing with the conversation, stopping and resuming at another time, or pausing and opting out of the study. The distress protocol was not enacted during the study. To avoid the transmission of Covid-19, safety precautions were followed during face-to-face contacts; arranging to meet outside or in a well-ventilated room, with social distancing, no physical contact, the wearing of masks and regular handwashing.

One researcher (EJ) with experience in data collection, transcription and translation listened to the audio recordings several times, noting all of research priorities and gaps in the current evidence-base on a MS Excel spreadsheet. In order to ensure reliable data analysis, two other researchers (SB and JM) examined data extraction and research questions of early interviews and provided input, after which a ‘long list’ of research questions was produced. For the process of reducing the long list to a shorter list of research questions, the research team worked together to group similar responses together, eliminated duplication and synthesized a single research question representing the focus of each group.

#### Round 2

Round 2 data collection was conducted online or by phone rather than face to face due to the Covid-19 pandemic. All the participants who completed Round 1 were invited to complete subsequent rounds. In Round 2, participants were asked to rate each item on the list generated from Round 1, on a scale of 1 to 5. The response choices for each question were: Very low/no importance (1), low importance (2), Moderate importance (3), Important (4), Very important (5). For academics, practice-based experts and advocates, this process was completed via an online survey using Qualtrics software. Each participant received an email with a link to the study research questions, together with an explanation of the process so far and instructions on how to complete the survey online. Participants were given two weeks to complete the survey. A reminder email and a phone follow-up were put in place after one week if no response was received. The rating exercise for patient / caregiver participants was conducted via telephone with the researcher entering the participant responses into the Qualtrics survey.

The data generated from Qualtrics was exported into a MS Excel sheet for data analysis. Questions that received 70% or more of the participants’ votes as either ‘important’ or ‘very important’ were identified for Round 3. Although a universally agreed proportion does not exist for the Delphi [18], a cut off of 70% agreement has been used in several other Delphi studies [30–32].

#### Round 3

Round 3 data collection was also conducted online, or by phone for the patients and carer participants. In this final Round, participants were asked to participate in a Qualtrics online survey to re-rank the reduced list of prioritized research resulting from Round 2. Each participant received a summary of the results of the previous round, including a list of research questions that had reached the threshold for retention, along with their individual responses and a summary of group responses from Round 2. All participants were informed that they could change the rankings they gave in the previous round when re-rating each research question in round 3. This informs the group members of the current status of their collective opinion and helps them to consider items that participants may have previously missed or thought unimportant [18]. This helped to increase concurrent validity while completing successive rounds of the questionnaire [18]. Once the surveys were completed, summary ranks for each question were determined on the basis of the numbers of participants rating each question as ‘important’ or ‘very important’.

### Ethical considerations

This study was approved by Nepal Health Research Council and ratified by the Research Ethics Committee of the Faculty of Health and Applied Sciences at the University of the West of England, Bristol.

## Results

### Expert panel information

Out of 50 invited experts, 42 participants completed Round 1 (response percentage 84.0%). Out of 42 participants, 38 of them participated in round 2 (retention rate = 90.4%) while 39 participants completed round 3 (92.8%). All the participants who completed round 1 were invited to rank questions in both round 2 and round 3. The sociodemographic characteristics of all the participants are shown in Table 2. The participants were aged 22–62 years (Mean ± SD = 39.4 ± 7.8, median = 38). There were eight psychiatrists, five psychologists, four psychiatric nurses, eleven researchers, seven advocates and seven patients or their family members. Almost all the participants were from Nepal (*n* = 41), one academic researcher lived in the United States. The participants had 1–33 years of experience in working in suicide prevention in Nepal.

**Table 2:** Characteristics of participants (*n* = 42)

Item	Category	N	%
Gender	Men	30	71.4
	Women	12	28.6
Age	Range	22–62	
	Mean ± SD	39.4 ± 7.8	
Area of expertise	Psychiatrists	8	19
	Psychologists	5	12
	Psychiatric nurses	4	9.5
	Researchers	11	26.1
	Advocates	7	16.7
	Suicide attempt survivors/family members	7	16.7

### Research questions

**Fig. 1:**
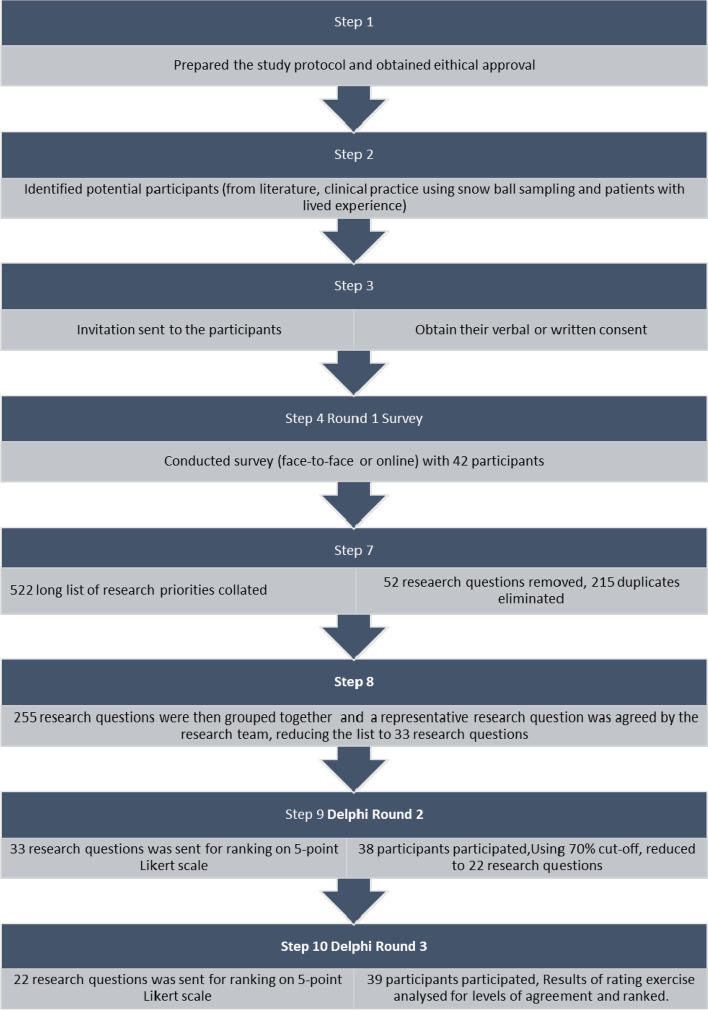
Delphi process

The 42 one-to-one interviews yielded a ‘long-list’ of 522 research questions at the end of Round 1. In order to achieve a manageable number of items for Round 2 [33], 52 research questions which were not directly related to suicide were removed for example, a) What are the barriers and facilitators for effective integration of mental health services? or b) What are the barriers for implementation of mental health action plans?. 215 duplicate research statements were removed and the remaining 255 research questions were then grouped together based on their similarity. For each cluster of remaining topics, a representative research question was agreed by the research team, reducing the list to 33 research questions at the end of Round 1. This process involved all members of the research team. These questions were sent to the participants for ranking in round 2. At the cut-off point of 70%, 22 research questions were ranked by the participants as either ‘important’ or ‘very important’. These remaining research questions were sent to the participants for re-ranking in round 3, together with information about their responses in round 2. Figure 1 shows the Delphi process. The final list of research questions after ranking in round 3 is shown in Table 3.

**Table3:** Research questions rated as ‘important’ and ‘very important

Questions	Round 2 Percentage of participants rating the question ‘important’ or ‘very important’ (%)	Round 3 Percentage of participants rating the question ‘important’ or ‘very important’ (%)
How can we improve the current underreporting of suicide data in Nepal?	82%	97%
What is the magnitude of the problem (completed suicides and attempted suicides) by geography, age, gender, and caste?	97%	95%
What are the underlying risk and protective factors (social, cultural, and economic) that contribute to suicide?	89%	95%
What are the enabling and impeding factors influencing the help-seeking behaviour of people experiencing suicidal thoughts?	97%	95%
What is the status and need for resources (human, equipment, and funding) at health facilities, police stations, and hotlines to manage patients who have suicidal ideation or have attempted suicide?	89%	95%
What kind of activities should be planned for suicide prevention in Nepal at the various levels of government (Federal system)?	79%	95%
Which groups of people are more vulnerable to attempting suicide in Nepal?	89%	92%
How can suicidal screening be strengthened in primary care settings, what tools should be used, and who should be screened?	84%	92%
What are the needs of families and carers who are trying to support someone who is at risk of suicide or died by suicide?	92%	90%
What is the status of psychiatric services (assessment, referral) in district hospitals for suicide attempt patients and what proportion of suicide attempt patients receive psychiatric services?	87%	90%
How can suicide attempt survivors and their family members be supported to advocate to reduce suicidal attempts and to improve awareness?	87%	90%
What are the pathways to care among people who have attempted suicide?	95%	87%
What kind of suicide prevention programme needs to be implemented for adolescents and children?	74%	87%
What are the effective suicide prevention interventions in low- and middle-income countries (systematic review)?	87%	85%
What are the support needs of suicide attempt survivors and their family members? (For example, safe space for disclosure, psychological support etc.)	87%	85%
What are the community's (adolescents', parents') and stakeholders' (police, health workers, key government officials') perceptions and attitudes toward suicide and those who died by suicide?	97%	82%
What are the lived experiences and mental health needs of suicide attempt survivors?	79%	82%
How do we improve compliance and engagement in healthcare follow up among people who have attempted suicide?	97%	82%
What should be included in a culturally appropriate community intervention to prevent suicide, and how should it be implemented?	79%	82%
What is the status of pesticide sales and purchase monitoring in Nepal, and how can it help prevent suicide?	71%	82%
What are the outcomes and rehabilitation need of people who have tried to end their life?	87%	77%
How can telepsychiatry be used to help people having suicidal ideation?	71%	62%

The prioritised research questions are observed to fall into three overarching topics. These are:

1. Studies to better understand the epidemiology of suicide in Nepal (e.g., burden, risk and protective factors, improvements in data, reporting system)
2. Studies to strengthen evidence-based practice on healthcare prevention and response service (e.g., improving access to support services, understanding health system resource requirements, screening services, mental health services)
3. Studies to better understand the needs of those at risk of self-harm and their families

## Discussion

This study is the first to assess perceptions of stakeholders on the potential research questions needed to inform and support suicide prevention in Nepal. The literature review conducted prior to this study suggested that there was limited research available from Nepal in this field. Our study has highlighted that studies across a range of key areas are considered important in order to support those at risk of suicide.

### Studies to better understand the epidemiology of suicide in Nepal

Participants prioritised studies that would improve the quality of suicide data, assess the burden of suicide, the factors associated with it, and the systems for collecting data. A study conducted in United States reported that the development of a comprehensive patient data collection system, such as real-time surveillance, death record linkage, and patient registries is an important first step to building evidence, and has the potential to facilitate later research to test various interventions [34]. In Nepal, as well as many South Asian countries, there is a lack of a comprehensive vital registration system [35]. National level suicide data are not systematically collected, and suicide mortality data are not reported by the WHO, but rather, are estimated [2, 13, 36, 37]. As suicide data are ‘owned’ by the police force in Nepal, there will need to be coordination and communication between the law enforcement and health systems in order to produce accurate estimates of suicide data [36]. Study participants were clear that putting robust systems in place to capture quality data is the fundamental challenge which needs to be addressed in Nepal. Future studies can focus on projects such as developing and piloting comprehensive surveillance systems for recording suicide and attempted suicides, and utilizing data from community surveillance systems, hospital and police records.

In the absence of accurate data on suicide in Nepal, our study experts expressed the need for a high quality nationally representative research programme on suicide and its causes. This resonates with studies from more developed countries that have called for studies of suicide prevention intervention in large samples, marginal groups and outpatients as research priorities to enhance patient safety [38]. In an Australian study reporting stakeholder’s views on future suicide prevention research, expert participants ranked evaluation studies assessing the efficacy of interventions, policies and programs most highly, followed by epidemiological studies of individual risk and protective factors [39]. In the current study, participants ranked the relative importance of research assessing risk factors (such as, previous suicidal attempt, family history of suicide etc.,) and protective factors (such as people’s capacity for resilience, hope and optimism) for suicide third on the final priority list. This implies that stakeholders in Nepal believed that knowing the national representative estimates of suicide rates in various groups and factors associated with it was an essential pre-requisite to developing and evaluating the most effective interventions.

Studies to strengthen evidence-based practice for healthcare prevention and response services A significant proportion of people who complete, attempt or consider suicide do not seek help from family members and health care facilities [39]. There may be many reasons for this, including beliefs about ineffective care, shame felt by people who have suicidal thoughts and their family members [40] and stigma against suicide and mental health issues appear to prevent people from using the limited resources available [6]. Studies have suggested that research is needed to clarify age and gender differences and the cultural and familial context of suicide bereavement, together with help seeking behaviours [41]. Consistent with these findings, participants in our study emphasised exploring the barriers and enablers for help seeking among people considering ending their life and their family members. Evidence on factors contributing to help seeking among these vulnerable group will guide the development of suicide prevention programs.

Examining the responses of the health and community service systems is an essential element of suicide prevention, as an ill-equipped health system will be unable to assess and manage people with suicidal thoughts or behaviours effectively [39]. Several components of healthcare provision such as trained human resource, timely referral, universal screening for those with suicidal thoughts etc., have been found to be associated with reduced suicide ideation and to mitigate deaths by suicide [34]. Despite the Ministry of Health in Nepal having had a mental health policy since 1997, including a vision to integrate mental health services into general health services and a Multisectoral Action Plan for the Prevention of Non-Communicable Diseases (2014–2020) that included mental health, progress has been slow. The mental health Gap Action Programme (mhGAP), promoting community-based mental health programmes, has been shown to reduce suicidal tendencies and encouraged establishment of support hotlines in limited parts of the country [6, 13]. However, the wider impact of this programme is yet to be determined [6]. Thus, studies assessing the status of such interventions and identification of the need for additional resources (human, equipment, and funding) at health facilities and police stations, appear warranted.

### Studies to better understand the needs of those at risk of self-harm, and their families

Those bereaved by suicide, whether family members or friends and colleagues, may experience a lasting impact of loss on their social life and on their physical and mental health [42]. Published literature highlights significant areas of need regarding interventions to be conducted after a suicide, including; a) what interventions work (for groups, individuals, online, outreach, etc.), b) for whom should they be developed (e.g. children, adolescents, older adults, workplace, prison, and other populations), and c) what outcomes should be measured (e.g., stigma, mental health, suicidality etc.) [41]. These recommendations were consistent with the findings of this study, with participants endorsing the need for research questions assessing the support needs of family members and ways to promote the implementations of appropriate interventions.

The funding and delivery of research questions prioritised in this study, across all three areas described, will enable the development and evaluation of culturally and contextually sensitive interventions for suicide prevention in Nepal.

### Limitations and strengths

To the best of our knowledge, this is the first Delphi study to collate research priorities for suicide prevention in Nepal. A key strength is that we included a wide range of stakeholders with different perspectives, including those with subjective expertise (patients and family members) and professional expertise (researchers, clinicians, advocates). This is in line with literature proposing that better quality and more broadly generalisable decisions are achieved through the process of achieving consensus in heterogenous groups [23]. Validity is also affected by the response rate [18] and retention rate, which in this study were very high. Employing face to face interviews where possible [18] and a quick turnaround time between questionnaires might have helped reduce attrition. The study provides a model for research prioritisation that may be useful for other communities to engage wide range of stakeholders.

Limitations of the study include the experts who took part were not asked about their awareness of existing research in the area Therefore, participants, particularly service users could have recommended research that had already been delivered. The wording of the questions included in the prioritisation rounds were kept as close as possible to that provided by the participants. Hence the research questions generated might need to be broken down or rephrased to enable them to be funded. Due to the Covid-19 pandemic, rounds 2 and 3 were conducted online or by phone rather than through group discussions which may have been preferable to encourage discussion and debate. However, our approach did provide participants with anonymity and confidentiality, which may have encouraged participation and engagement, and prevented dominance by influential individuals or group pressure that may otherwise have occurred [25]. Our study recruited service users and family members through one healthcare facility. We acknowledge that people with suicidal thoughts and their families not using this service may have been able to offer alternative views.

### Implications and recommendations

The findings from this study will help researchers, healthcare professionals, and policymakers prioritise funding strategies relating to suicide prevention. The findings may guide collaborations comprising governmental, non-governmental, health workers and people with lived experience to work together to generate scientific evidence. More importantly, studies that delve into the outcomes associated with exploring ways to improve suicide reporting, as well as assessing the burden and factors associated with suicide have much to offer in increasing the understanding of this area.

## Conclusion

To date, suicide has had little attention as a public health problem in Nepal. Despite a growing number of studies, this is the first expert consensus study to identify the top research priorities that should be addressed in future research so as to prevent and control suicides in Nepal. The study reports the views of people who are experts in the field of mental health & suicide and suicide survivors, offering an important contribution to efforts to improve suicide prevention in Nepal. A priority driven approach to research on suicide prevention has the potential to result in an evidence-base to offer authoritative guidance to those who devote their working lives to suicide prevention.

## Supplementary information

**Additional file 1.**

## Data Availability

All data generated or analysed during this study are included in this published article [ and its supplementary information files].

## References

[1] World Health Organization. National suicide prevention strategies: Progress, examples and indicators. Geneva: World Health Organization; 2018.

[2] World Health Organization. Suicide in the World: Global Health Estimates. Geneva: World Health Organization; 2019.

[3] The Lancet: Suicide prevention: steps to be taken. Lancet. 2012;379(9834):2314. 10.1016/S0140-6736(12)61000-9 22726499

[4] Vijayakumar L, Chandra PS, Kumar MS, Pathare S, Banerjee D, Goswami T, et al. The national suicide prevention strategy in India: context and considerations for urgent action. Lancet Psychiatry. 2021;9:160–8. 10.1016/S2215-0366(21)00152-8 34895477

[5] Jordans M, Rathod S, Fekadu A, Medhin G, Kigozi F, Kohrt B, Luitel N, Petersen I, Shidhaye R, Ssebunnya J (2018). Suicidal ideation and behaviour among community and health care seeking populations in five low-and middle-income countries: a cross-sectional study. Epidemiol Psychiatr Sci.

[6] Marahatta K, Samuel R, Sharma P, Dixit L, Shrestha BR (2017). Suicide burden and prevention in Nepal: the need for a national strategy. WHO South East Asia J Public Health.

[7] World Health Organization. Preventing suicide: a global imperative. Geneva: World Health Organization; 2014.

[8] World Health Organisation. Suicide Worldwide in 2019: global health estimtes. Geneva: World Health Organization; 2021.

[9] Silwal BS,  Pant PR, Gurung YB (2021). 2E. 003 Burden of suicide in Nepal: an analysis of police records. Inj Prev.

[10] Bhatta S, Mytton J, Joshi E, Bhatta S, Adhikari D, Manandhar SR, Joshi SK (2021). Development and Evaluation of a Community Surveillance Method for Estimating Deaths Due to Injuries in Rural Nepal. Int J Environ Res Public Health.

[11] Pant PR, Banstola A, Bhatta S, Mytton JA, Acharya D, Bhattarai S, Bisignano C, Castle CD, Dhungana GP, Dingels ZV (2020). Burden of injuries in Nepal, 1990–2017: findings from the Global Burden of Disease Study 2017. Inj Prev.

[12] Mytton J, Bhatta S, Thorne M, Pant P (2019). Understanding the burden of injuries in Nepal: A systematic review of published studies. Cogent Med.

[13] Thapaliya S, Sharma P, Upadhyaya K (2018). Suicide and self harm in Nepal: A scoping review. Asian J Psychiatr.

[14] Sharma B, Kim H-Y, Kim J-R, Nam E-W (2018). A Study on Suicide and Suicide Prevention Policies in Nepal. Korean Public Health Res.

[15] Silverman MM, Pirkis JE, Pearson JL, Sherrill JT (2014). Reflections on expert recommendations for US research priorities in suicide prevention. Am J Prev Med.

[16] Vijayakumar L (2010). Indian research on suicide. Indian J Psychiatry.

[17] Saini P, Clements C, Gardner KJ, Chopra J, Latham C, Kumar R, Taylor P: Identifying suicide and self-harm research priorities in north west England: A Delphi study. Crisis: The Journal of Crisis Intervention and Suicide Prevention. 2021. 10.1027/0227-5910/a000757 33475010

[18] Hasson F, Keeney S, McKenna H (2000). Research guidelines for the Delphi survey technique. J Adv Nurs.

[19] Viergever RF, Olifson S, Ghaffar A, Terry RF (2010). A checklist for health research priority setting: nine common themes of good practice. Health Res Policy Syst.

[20] Wang DWL, Colucci E (2017). Should compulsory admission to hospital be part of suicide prevention strategies?. BJPsych bulletin.

[21] World Health Organization. Live life: an implementation guide for suicide prevention in countries. Geneva: World Health Organization; 2021.

[22] Bond KS, Cottrill FA, Blee FL, Kelly CM, Kitchener BA, Jorm AF (2019). Offering mental health first aid to a person with depression: a Delphi study to re-develop the guidelines published in 2008. BMC Psychol.

[23] Jorm AF (2015). Using the Delphi expert consensus method in mental health research. Aust N Z J Psychiatry.

[24] Skulmoski GJ, Hartman FT, Krahn J (2007). The Delphi method for graduate research. J Res Technol Educ.

[25] Wilson A, Averis A, Walsh K (2003). The influences on and experiences of becoming nurse entrepreneurs: a Delphi study. Int J Nurs Pract.

[26] Vella K, Goldfrad C, Rowan K, Bion J, Black N (2000). Use of consensus development to establish national research priorities in critical care. BMJ.

[27] Powell C (2003). The Delphi technique: myths and realities. J Adv Nurs.

[28] Goodman CM (1987). The Delphi technique: a critique. J Adv Nurs.

[29] Curtis E, Drennan J (2013). Quantitative health research: issues and methods: issues and methods.

[30] Colucci E, Kelly CM, Minas H, Jorm AF, Chatterjee S (2010). Mental Health First Aid guidelines for helping a suicidal person: a Delphi consensus study in India. Int J Ment Health Syst.

[31] Setkowski K, Van Balkom AJ, Dongelmans DA, Gilissen R (2020). Prioritizing suicide prevention guideline recommendations in specialist mental healthcare: a Delphi study. BMC Psychiatry.

[32] Sumsion T (1998). The Delphi technique: an adaptive research tool. Br J Occup Ther.

[33] Kelly CM, Jorm AF, Kitchener BA (2009). Development of mental health first aid guidelines for panic attacks: a Delphi study. BMC Psychiatry.

[34] Ahmedani BK, Vannoy S (2014). National pathways for suicide prevention and health services research. Am J Prev Med.

[35] UNICEF ROSA (2019). Status of civil registration and vital statistics in South Asia countries, 2018. Registration of births, deaths and marriages.

[36] Hagaman AK, Maharjan U, Kohrt BA (2016). Suicide surveillance and health systems in Nepal: a qualitative and social network analysis. Int J Ment Health Syst.

[37] Jordans MJ, Kaufman A, Brenman NF, Adhikari RP, Luitel NP, Tol WA, Komproe I (2014). Suicide in South Asia: a scoping review. BMC Psychiatry.

[38] Dewa LH, Murray K, Thibaut B, Ramtale SC, Adam S, Darzi A, Archer S (2018). Identifying research priorities for patient safety in mental health: an international expert Delphi study. BMJ Open.

[39] Niner S, Pirkis J, Krysinska K, Robinson J, Dudley M, Schindeler E, De Leo D, Warr D (2009). Research priorities in suicide prevention: A qualitative study of stakeholders’ views. Australian e-Journal for the Adv Ment Health.

[40] Ahmedani BK, Kubiak SP, Kessler RC, de Graaf R, Alonso J, Bruffaerts R, Zarkov Z, Viana MC, Huang Y, Hu C (2013). Embarrassment when illness strikes a close relative: A world mental health survey consortium multi-site study. Psychol Med.

[41] Andriessen K, Dransart DAC, Cerel J, Maple M (2017). urrent postvention research and priorities for the future. Crisis.

[42] Maple M, Pearce T, Sanford R, Cerel J, Dransart DAC, Andriessen K: A systematic mapping of suicide bereavement and postvention research and a proposed strategic research agenda. Crisis. 2017. 10.1027/0227-5910/a000498 29256269

