# Supplementary material for: Identification of research priorities for suicide prevention in Nepal: a Delphi study
Source: BMC Psychiatry. 2022 Jun 25;22:429. doi: 10.1186/s12888-022-04074-z (PMC9233798; doi:10.1186/s12888-022-04074-z)
Supplement: Supplementary file 1 — Additional file 1. [file 12888_2022_4074_MOESM1_ESM.pdf]

Round 1: Questionnaire for Researchers/Practitioners/Advocates

Participant's Identification Number:

S. No.	Questions	Response
**Section I**		
**1.**	**Name**	…………………………………………
**2.**	**Age**	…………………………………………
**3.**	**Gender**	a. Male<br>b. Female<br>c. Others (please specify) ………………
**4.**	**Permanent Address**	Province: …………………… District: …………………… City: …………………………
**5.**	**Temporary Address (leave blank if not relevant)**	Province: …………………… District: …………………… City: …………………………
**6.**	**Profession**	a. Researcher<br>b. Clinician<br>c. Advocates<br>d. Academician<br>e. Others (please specify) ………………
**7.**	**How long have you been involved in the field of suicide prevention?**	………. years<br>………. months
**8.**	**Name of current Institutions/Organizations**	……………………………………………

Section 2		
**9.**	**In your view, what is the status, causes/risk factors of suicide and self-harm in Nepal?**	
**10.**	**What is working well for suicide prevention in Nepal? (Aspects of health care delivery services, awareness, government plan and programs, community)**	

**11.**	**What is not working well/challenges do you face while conducting your research/ practice with people who attempt to commit suicide? ((Aspects of health care delivery services, awareness, government plan and programs, community)**	
**12.**	**What additional information/ evidence would help your research or you work supporting people considering taking their life?**	

13.	Can you provide 5-10 research questions that you think would improve the evidence to prevent suicide and attempted suicide in Nepal over the next 5 years.	i.<br><br>ii.<br><br>iii.<br><br>iv.<br><br>v.<br><br>vi.<br><br>vii.<br><br>viii.<br><br>ix.<br><br>x.

**Section 3**		
**12.**	**Can you let us know about any other organization or people who could be potential participants for our study?**	a. Yes (if yes please go to Q.No.13)<br>b. No (if no please go to Q.no. 14)
**13.**	**Please provide us with details**	Name:<br>Professional Title:<br>Organization<br>Contact Details
		Name:<br>Professional Title:<br>Organization<br>Contact Details
		Name:<br>Professional Title:<br>Organization<br>Contact Details
**14.**	**Would you be interested to be a part of Suicide Prevention Advisory Group in future?**	**Yes**<br>**No**

Thank you!

Round 1: Questionnaire for patients, care givers and family member participants.

Participant's Identification Number:

S. No.	Questions	Response
**Section I**		
**1.**	**Name**	…………………………………………
**2.**	**Age**	…………………………………………
**3.**	**Gender**	a. Male<br>b. Female<br>c. Others (Please specify)….
**4.**	**Are you someone who has previously attempted to harm themselves?**	a. Yes<br>b. No<br>If Yes, how long has it been?..........years…………………months
**5.**	**If No, please tell us your relationship with the person who tried to harm themselves?**	a. Caregiver<br>b. Family member<br>c. Friend<br>d. Others (Please specify) …………
**6.**	**What is your Permanent Address**	Province: …………………… District: …………………… City: …………………………
**7.**	**What is your Temporary Address (leave blank if not relevant)**	Province: …………………… District: …………………… City: …………………………
**Section 2**		
**8.**	**What do you think are the most important things that researchers or doctors and nurses**	

	**should study or do in order to help people who are thinking of harming themselves?**	
**9.**	**At individual/family level, how do you think we can reduce or prevent people from harming themselves?**	
**10.**	**At community level, how do you think we can reduce or prevent people from harming themselves?**	

**11.**	**At national level, how do you think we can reduce or prevent people from harming themselves?**	

Thank you!
